# The practices, challenges and recommendations of South African audiologists regarding managing children with auditory processing disorders

**DOI:** 10.4102/sajcd.v63i1.132

**Published:** 2016-06-09

**Authors:** Claire Fouché-Copley, Samantha Govender, Nasim Khan

**Affiliations:** 1 Discipline of Audiology, University of KwaZulu-Natal, South Africa

## Abstract

Audiologists managing children with auditory processing disorders (APD) encounter challenges that include conflicting definitions, several classification profiles, problems with differential diagnosis and a lack of standardised guidelines. The heterogeneity of the disorder and its concomitant childhood disorders makes diagnosis difficult. Linguistic and cultural issues are additional challenges faced by South African audiologists. The study aimed to describe the practices, challenges and recommendations of South African audiologists managing children with APD. A quantitative, non-experimental descriptive survey was used to obtain data from 156 audiologists registered with the Health Professions of South Africa. Findings revealed that 67% screened for APD, 42% assessed while 43% provided intervention. A variety of screening and assessment procedures were being administered, with no standard test battery identified. A range of intervention strategies being used are discussed. When the relationship between the number of years of experience and the audiologists’ level of preparedness to practice in the field of APD was compared, a statistically significant difference (*p* = 0.049) was seen in that participants with more than 10 years of experience were more prepared to practice in this area. Those participants having qualified as speech-language therapists and audiologists were significantly more prepared (*p* = 0.03) to practice than the audiologists who comprised the sample. Challenges experienced by the participants included the lack of linguistically and culturally appropriate screening and assessment tools and limited normative data. Recommendations included reviewing the undergraduate audiology training programmes, reinstituting the South African APD Taskforce, developing linguistically and culturally appropriate normative data, creating awareness among educators and involving them in the multidisciplinary team.

## Introduction

The only currently available international prevalence of auditory processing disorders (APD) in the paediatric population is estimated to be between 2% and 5% (Chermak, Silva, Nye, Habrouck & Musiek, 2007). Determining the prevalence of APD in children remains a challenge, as children presenting with mild symptoms of APD may go undetected because of the subtle symptoms, while conditions such as attention deficit hyperactivity disorder (ADHD) and learning disorders (LD) can influence differential diagnosis (Baldry & Hind, 2008). APD, or alternatively Central Auditory Processing Disorders (CAPD), is a particular type of hearing difficulty that occurs along the auditory pathway and involves the Central Auditory Nervous System (CANS), typically presenting with normal hearing sensitivity or a normal audiogram (American Academy of Audiology [AAA], 2010; British Society of Audiology [BSA], 2011). APD is idiopathic in nature, and some authors suggest that it may even have no anatomical site of pathology (AAA, 2010). The difficulties experienced by children with APD are their inability to separate meaningful auditory information (speech) from non-meaningful information (background noise) which obscures the auditory messages conveyed to the brain (AAA, 2010). APD occurs across all ages and typically manifests as poor attention to auditory stimuli, difficulty attending to foreground acoustic information in the presence of background noise, difficulty with auditory memory and delayed receptive language development (Bantwal, 2011; Medwetsky, 2011; Witton, 2010).

Several classification profiles further complicate the diagnosis and management of the disorder. Initially stemming from a single entity, APD branches into several diverse pathways, manifesting as listening, reading, spelling and even attention difficulties, creating further detriment in the ability to cope with everyday activities (Sharma, Purdy & Kelly, 2012). The implications for academic progress can be considerable, with the listener becoming frustrated, irritable and often unable to follow instruction, which also impacts on their social and emotional development (McMahon, 2011; Witton, 2010). Therefore, the multifaceted nature of APD calls for effective differential diagnosis from a team comprising diverse professionals. A valuable member of this team includes the audiologist and speech-language therapist and audiologist (STA). Assessing and managing APD is part of the scope of practice for audiologists (HPCSA, 2008); however, based on the literature, they lack confidence in managing APD because of several challenges (Baldry & Hind, 2008; The Canadian Interorganizational Steering Group for Speech-Language Pathology and Audiology [CISG], 2012; Logue-Kennedy et al., 2011).

These challenges relate to determining the anatomical origin of APD, which affects defining the disorder and results in inadequately formulated guidelines regarding appropriate test batteries, as well as a lack of management options (Ferguson, 2014). In addition, South African audiologists confront the challenges relating to linguistic and cultural diversity, as the country has eleven official languages and various cultural groups.

The current status of service delivery within the South African context further places constraints on APD services offered by audiologists. Most audiology services are unequally distributed in urban areas and are provided mainly through the private sector (Pascoe & Norman, 2011). According to the Department of Health, KwaZulu-Natal (DOH, 2010), more than half of the population live in rural settlements, suggesting that these individuals have limited access to basic services. An increase in non-communicable diseases, such as the HIV/AIDS and Tuberculosis (TB), further exacerbates poverty and under-development (Bradshaw et al., 2003). Healthcare resources are therefore directed towards fighting disease, while persons with communication disorders receive low priority and limited resource allocation (Pascoe & Norman, 2011). Given the association between disease and hearing loss, audiologists, especially those working within the public health sector, are overwhelmed with the burgeoning demands for service delivery with the focus being placed on the management of more commonly occurring conditions. It is therefore, a challenge to provide audiology services to children presenting with APD and other auditory pathologies.

The 1802 audiologists and STAs registered with the HPCSA in 2013 provided services to a culturally and linguistically diverse population of approximately 54 million people (Statistics South Africa, 2014). However, the majority of the audiologists in South Africa either speak English or Afrikaans (Pascoe & Norman, 2011), therefore creating a mismatch between audiologists and their linguistically diverse clients in terms of language, which further impacts on service delivery (Pascoe, Rodgers & Norman, 2013). The majority of the South African population (23%) are first-language isiZulu speakers, whilst only 14% are first-language English speakers (Statistics South Africa, 2014). Due to the lack of linguistically appropriate assessment tools, there are few standardised normative data available, therefore influencing the reliability and validity of screening and assessment measures (Ferguson, 2014). This impacts on appropriately diagnosing and managing children presenting with APD.

In an effort to address some of the above-mentioned challenges, the South African Taskforce was established in 2001. Its aim was to establish an appropriate test battery for both the fluent first-language, English-speaking child and one who is non-proficient in English, that is second-language English speaker. As a result, the South African Low Linguistically Loaded CAPD Test Protocol was created to cater for individuals with a basic understanding of the English language (Saleh, Campbell & Wilson, 2003). However, it has been challenging to apply within the South African population because several of the APD tests recommended were developed in the United States and influenced by foreign data with the linguistic load disadvantaging children with different dialects (Ferguson, 2014).

The South African Taskforce (2001) document recommends two dichotic tests (one being linguistically loaded and another non-linguistically loaded), one monaural low redundancy speech test, one temporal pattern test and one binaural interaction test. However, the two popular dichotic tests, namely, the Dichotic Digits Test and the Frequency (Pitch) Pattern Test, were developed over 40 years ago and do not provide adequate supporting documentation and normative data, while the Dichotic CV test is also not appropriate for younger children or for populations with a high degree of linguistic diversity and is proven to show a great degree of variability in school-aged children (Bellis, 2003). The above challenges therefore question the reliability of some of the common assessment tools available to audiologists, who may therefore have to rely on several assessment tools, making the diagnosis of APD a challenge. It is important that appropriate screening and diagnostic protocols are used in order to facilitate detection, which in turn can favour appropriate intervention.

Bellis (2003) recommends that in order for intervention to be successful, a ‘tripod’ approach should be implemented, which includes a combination of environmental modifications, compensatory strategies and direct skills remediation. Therefore, it is necessary to identify the nature of the APD before determining the way forward in terms of selecting appropriate intervention strategies or making the necessary referrals (Bellis, 2003). Bellis (2003) further emphasises that the interdependency between several practitioners, such as the speech-language therapist, psychologist, social worker, teacher, physician and parent, is significant in determining the child’s difficulties, strengths and progress. The audiologist plays a significant role in the management of the APD programme and therefore needs to ensure collaboration among the different team members (Bellis, 2003).

By understanding the challenges experienced by audiologists, areas requiring attention will be highlighted that have been overlooked, thereby facilitating the development of contextually appropriate guidelines and protocols to meet the professions demands. This will enable audiologists to become more equipped to provide a service of high quality and that which complies with ethical standards (Knudsen et al., 2012). Further research in the area may also provide information on how to create more cost-effective and realistic solutions to suit the South African context. It is also of benefit to determine how audiologists in South Africa are practicing in the area of APD and how various challenges are being accommodated for.

The study therefore aimed to determine the practices, challenges and recommendations of South African audiologists regarding managing children with APD. The study included an open-ended question, allowing participants to report on their own personal opinions of challenges restricting them from practicing in the area of APD and to provide recommendations to improve APD services in South Africa. Such information can be used in policy formulation and to improve service delivery.

## Research method and design

### Aims

The study aimed to describe the practices, challenges and recommendations of South African audiologists regarding managing children with APDs. This was done by surveying the practices and challenges of audiologists regarding the screening, assessment and intervention of children with APD. In addition, the study outlined the recommendations provided by the study participants with regards to managing children with APD. The study was situated within a positivist paradigm, with a quantitative, non-experimental descriptive survey design being used.

### Sample

A total of 189 of 1802 audiologists and STAs accessed from the national HPCSA register for 2013 consented to participate in the study. A total of 156 questionnaires were considered for analysis yielding a response rate of 8.6%. The participants were accessed from two separate HPCSA registers namely, the audiology and speech therapy and audiology registers. The participants predominantly practiced in Pretoria, the Western Cape and KwaZulu-Natal, with fewer responses from the other six provinces. The common languages spoken by the audiological caseload of the participants included English, Afrikaans and isiZulu. There appeared to be an equal percentage of responses from audiologists and STAs. Of the 156, 49% were in private practice, 19% were at public health sector and 17% were at schools. Most of the participants had an undergraduate Bachelor degree (BA, 80%, *n* = 125), 29 (19%) had a Masters (MA) and two had completed their doctorate (PhD, 1%). The details are reflected in Table 1.

**TABLE 1:** Demographical profile of study participants.

Work experience in years and qualification details	BA number (80%)†	MA number (19%)‡	PhD number (1%)§	Total number	Total %
**Years of experience**					
0–5	38 (30%)	3 (10%)	0	41	26
6–10	31 (25%)	9 (31%)	0	40	25
11–15	17 (14%)	3 (10%)	0	20	13
> 15	39 (31%)	14 (48%)	2 (100%)	55	35
**Year of qualification**					
After 2006	51 (41%)	6 (21%)	0	57	37
2000–2005	26 (21%)	7 (24%)	0	33	21
1990–1999	25 (20%)	9 (31%)	0	34	22
1980–1989	19 (15%)	5 (17%)	1 (50%)	25	16
Prior to 1989	4 (0%)	2 (7%)	1 (50%)	7	5
**Institute**					
University of Cape Town	16 (13%)	7 (24%)	0	23	15
Stellenbosch University	9 (7%)	2 (7%)	0	11	7
University if Pretoria	51 (41%)	8 (28%)	2 (100%)	61	40
University of Kwazulu-Natal	26 (21%)	3 (10%)	0	29	19
University of Witwatersrand	20 (16%)	9 (31%)	0	29	19
Other	3 (2%)	-	0	3	2

† *n* = 125;

‡ *n* = 29;

§ *n* = 2

### Data collection method

A questionnaire survey was used to collect the data. Permission was granted by the APD Ireland Research Group, to adapt their questionnaire entitled: Current and Future Service Provision for Children with Auditory Processing Disorder in Ireland (APD Ireland Research Group, 2008), with the number and sequences of the questions being amended to match the objectives of the current study. The APD Ireland Research Group (2008) questionnaire comprised two phases, the first phase being quantitative and the second phase being qualitative. The current research only used and adapted the first phase of the questionnaire and consisted of 11 questions (38%), which were reworded to suit the context of the study, while an additional 18 questions (62%) were formulated based on extensive literature and research studies performed in the field of APD such as American Speech-Language and Hearing Association (ASHA), 2005), AAA (2010), Bellis (2003), Ferguson (2014) and Elsisy (2013). Seven of the open-ended questions from the previous study were converted into closed-ended questions. An open-ended question was included in order to obtain participants’ recommendations.

### Data collection procedure

All audiologists and STAs were given an equal opportunity to participate in the study. The questionnaire, information letter and consent form with an electronic link to the online survey site, Survey Monkey, were posted to all the audiologists and STAs on the HPCSA register. The participants were given a choice of either responding by means of an electronic questionnaire or a hardcopy questionnaire. An acknowledgment of consent had to be completed on the Survey Monkey electronic questionnaire before proceeding with the questionnaire. Respondents were given a time frame of 12 days to complete the questionnaire.

### Reliability and validity

A pilot study was conducted, which revealed that the electronic platform used to collect data, together with the questionnaire and related documents, was easy to access and complete. An adapted questionnaire was used to improve the reliability of the data collection tool. Additional questions included in the survey were formulated based on extensive literature and research studies performed in the field of APD.

## Ethical considerations

Ethical approval was obtained from the Humanities and Social Sciences Research Ethics Committee of the University of KwaZulu-Natal (Certification Number: 1186567), and permission was granted from the HPCSA to access the member’s list. The current study follows the recommendations provided by Alcser, Antoun, Bowers, Clemens and Lien (2011), which supports the participant’s rights of free will, privacy and confidentiality. Permission was obtained from the APD Ireland Group (2008) before adapting the questionnaire. Participant information was profiled by using research participant numbers and coded accordingly. Participation in the study was voluntary, there were no risks and the participants were entitled to withdraw from the study at any stage. The researcher completed an online ethics course to ensure that all ethical issues pertaining to the study had been addressed.

## Results and discussion

The results are presented with respect to the study’s two objectives, the first being to describe the practices and challenges of audiologists regarding the screening, assessment and intervention of children with APD and the second objective was to describe the recommendations provided by participants.

### Objective one

#### Screening

**FIGURE 1:**
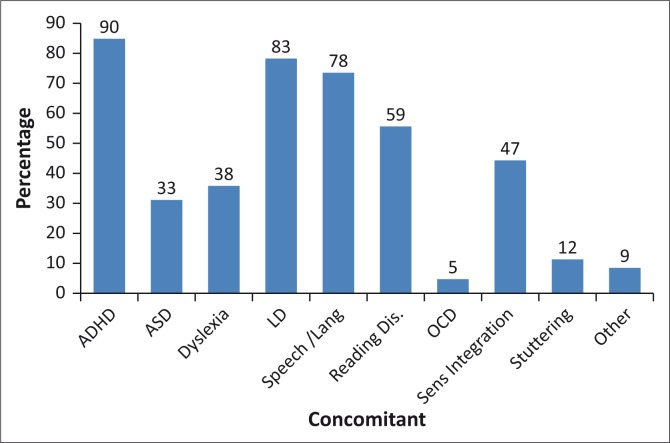
Common concomitant childhood disorders associated with auditory processing disorders.

Of the 156 respondents, 60% (*n* = 93) reported to have screened children for APD, with 40% (*n* = 37) indicating that they did not follow any formal guidelines and/or policies related to screening. Common guidelines that were used by audiologists included the RSA CAPD Taskforce (2001) document (29%, *n* = 27), the ASHA) (2005) document (31%, *n* = 28), the Bellis (2003) guidelines (33%, *n* = 30) and the AAA (2010) guideline. The school teacher was the primary referral source to audiologists (52%, *n* = 69), with 96% (*n* = 89) of referrals consisting of children presenting with poor academic performance at school, 64% (*n* = 59) inattentiveness and/or distractibility and 45% (*n* = 42) poor speech and language development. In the current study, the most common concomitant childhood disorders often associated with their APD caseload were ADHD, LD and speech-language conditions as illustrated in Figure 1.

The participants used several combinations of formal and informal screening tools, such as the CHAPPS, Fisher’s and SCAN C, which are illustrated in Table 2.

**TABLE 2:** Common screening tools administered by South African audiologists.

Screening tool	Formal or informal	%	*n*
Children’s Auditory Processing Performance Scale	Informal	48	45
Fisher’s Auditory Processing Checklist	Informal	29	27
SCAN:C	Formal	28	26
Screening Instrument for Targeting Educational Risk	Informal	16	15
SCAN:3C	Formal	16	15
SCAN:A	Formal	15	14
Auditory Continuous Performance Test	Formal	12	11
Listening Inventory for Education Checklist	Informal	11	10

#### Assessment

Of the 156, 42% (*n* = 66) assessed children for APD, the four common assessment tools being the low linguistically loaded Dichotic Digits Test (32%, *n* = 24), the Frequency (Pitch) Pattern Test (31%, *n* = 23) the Low-Pass Filtered Speech Test (28%, *n* = 21) and the linguistically loaded Dichotic Sentence Test (25%, *n* = 19). Twenty percent (*n* = 15) of the participants administered electrophysiological tests, while additional tests included those that were more speech and language driven.

#### Intervention

A total of 43% (*n* = 67) participants provided interventions for children presenting with APD. Of the 67 who provided interventions, 83% (*n* = 55) used preferential seating as an intervention strategy, 80% (*n* = 54) emphasised the importance of gaining the child’s attention before speaking and 77% (*n* = 52) recommended repeating and rephrasing the instruction. A total of 61% (*n* = 41) recommended the use of frequency modulated (FM) systems, while 51% (*n* = 34) recommended compensatory strategies. Little focus was placed on direct skills remediation and auditory training. There appeared to be a statistically significant relationship between the number of years of experience and the provision of intervention for APD, as 59% (*p* = 0.00) with more than 10 years of experience, and only 31% with less than 10 years of experience provided interventions. Forty-six percent (*n* = 72) of the participants provided onward referral to other practitioners, the most common being made primarily to occupational therapists (56%, *n* = 40), psychologists (51%, *n* = 37) and a second audiologist (51%, *n* = 37).

An open-ended question was included, where the participants could document further concerns with regards to managing children with APD, of whom 51% (*n* = 79) indicated their challenges. The pass/fail criterion provided by certain screening tools was a concern to the participants (49%, *n* = 39), while tools were considered either ‘too complicated’, ‘too long to administer’ or ‘too boring for children’. Participants further indicated that the time taken to assess the child and write a full APD report could not be justified for the amount charged for an APD evaluation, which most medical aids do not cover (5%, *n* = 4). Therefore, audiologists may potentially avoid practicing in the area of APD and rather refer elsewhere. They reported a lack of standardised screening and assessment tools with minimal supporting documentation and/or normative data. Cultural and linguistic issues were also a common concern, as many South African children are not affluent in English and are disadvantaged when using the above measures to asses for APD. In addition, there was a perceived lack of collaboration between the audiologist and the SLT (6%, *n* = 5). The different roles between the audiologist and speech therapist in identifying and managing APD were unclear to the participants. They were unfamiliar with which practitioner to refer children to, potentially resulting in delayed intervention or none being provided. Eighteen percent (*n* = 14) of the participants indicated that they may have benefitted from additional theoretical and clinical preparation during their undergraduate programme, while others were restricted by budget constraints and hospital policies to continue with training and development in this area (19%, *n* = 15). A lack of adherence from parents and teachers to comply with the assessment and intervention programmes was another concern raised. A few of the participants (13%, *n* = 10) attributed this to the lack of awareness among teachers, parents and other practitioners. Informal screening questionnaires were often delivered incomplete, while teachers did not always see the need for classroom modifications, particularly when accommodating larger classes. There appears to be a concern regarding the lack of communication between the various members involved in the multidisciplinary team who need to be involved in managing children with APD.

## Discussion

The findings of the present study revealed that a variety of test procedures are being administered by audiologists to manage children with APD. These findings are due to linguistic and cultural variations, a lack of standardised protocols, limited normative data and minimal supporting documentation to secure a diagnosis. Audiologists are not familiar with who to refer to or how to manage children with APD, despite it being part of their scope of practice. As a result, children who do not have an APD are either over referred, or worse, children presenting with an APD are not referred at all and go undiagnosed.

The multicultural nature of South Africa calls for the sensitivity of clinical practice to accommodate the South African context, which is often challenging for the audiologist.

Vaughn, Jacquez and Baker (2009) suggested that in order for healthcare providers to follow an ecological approach to assessment and intervention, additional training regarding culturally sensitive practice is crucial. According to the AAA (2010) and the ASHA (2005) document, both verbal and non-verbal assessment tools are recommended in order to paint a clearer picture of the CANS, the underlying processes and the possible location of dysfunction. However, despite having these tools available, most audiological assessment tools for APD are not suitable for the linguistically rich context of South Africa, and the country’s 11 different languages, making the assessment and diagnosis of APD challenging. APD tests are also not standardised to suit the South African population, questioning the reliability and validity of the screening and assessment measures within the South African context (Saleh et al., 2003).

The contextually specific challenges of South Africa – which include poverty, malnutrition, limited access to medical and educational facilities, increase in burden of disease, culturally and linguistically inappropriate test materials – all restrict effective service delivery. These findings were similar to a study performed in India, which is also a linguistically diverse and enriched country. In addition, India, with a population of 1.2 billion, consisting of 22 different languages and served by 1750 audiologists registered with the Indian Speech and Hearing Association, presents challenges similar to those in South Africa (Bantwal, 2011). The challenges include linguistic and cultural issues, similar to the present study. These statistics differ to those of a survey reported on the United States, which indicated that more than 80% of the population spoke English as their first language (United States Census Bureau, 2011). Based on the above findings, practitioners need to consider the linguistic background of the client before administering the test battery.

There is considerable criticism about the many informal and formal screening tools administered, with the CHAPPS (the most commonly used screening tool) and the Screening Instrument for Targeting Educational Risk not serving as clear indicators as to whether a full diagnostic APD evaluation is warranted rather only highlighting areas of the child’s weaknesses (Emanuel, 2002). The Fisher’s Auditory Problems Checklist was criticised for its limited categorical organisation (Wilson et al., 2011). Despite the SCAN being one of the most popular formal screening tools administered on an international level, with 50% sensitivity, it has been criticised due to its poor test–retest reliability, its linguistically loaded nature (Elsisy, 2013) and its inability to adapt to other cultures and languages (Logue-Kennedy et al., 2011). Bellis (2003) also cautioned that children with APD may pass the SCAN tests and that it should therefore be administered in conjunction with other screening measures. As identified in the current study, audiologists are administering several screening tools before recommending an audiological evaluation due to the lack of test reliability and validity in using one screening tool.

The findings of the present study are congruent with that of the study conducted by Logue-Kennedy *et al.* (2011), where more than half of the participating audiologists indicated that very few screening tools were used for APD, often due to a lack of experience in the area. The majority of the audiologists in the above-mentioned study did not feel competent to practice in the area of APD, more so than the half of educational psychologists that comprised the sample, despite audiologists being the primary practitioner to manage children with APD (HPCSA, 2008). Therefore, audiologists offered advice about managing APD based on their observation and information from a variety of sources, rather than administering APD tests (Logue-Kennedy et al., 2011).

In a study conducted by Chermak, Traynham, Seikel and Musiek, (1998), which aimed to determine audiologists’ knowledge and use of APD assessment tools in the United States, less than half of the participants felt competent to administer APD assessments. Eighty percent of the participants received training in the field of APD, yet less than half felt confident in the area and reported having only spent as little as an average of 3 hours clinical contact with the APD population throughout their training. A study performed by the Canadian Interorganizational Steering Group for Audiology and Speech-Language Pathology (CISG, 2012) mirrored these findings, where less than half of the audiologists assessed children for APD. Those participants not providing APD services reported that they tended to prioritise other audiological disorders and focused on services such as hearing aid fittings, which were considered a priority over APD. The language used on tests appears to be a significant barrier, as the tests should be able to reflect valid sensitivity and specificity and account for subject variables, such as higher order functions, chronological age and language, which are often not documented in the common assessment tools of today (Elsisy, 2013). As a result, audiologists are selecting a range of tests and creating their own test batteries, potentially allowing for gaps in APD assessment. Speech and language assessment tools are also often being administered to account for these gaps, which are not always effective in comprehensively assessing and diagnosing APD, unless the auditory signal of the test material is degraded or altered in any way (Vanniasegaram, Cohen & Rosen, 2004).

Bellis (2003) emphasised that audiologists should not screen a child for APD if they cannot support the child with the necessary assessment procedures and/or intervention tools. While it is acknowledged that intervention strategies should be geared to each child’s specific needs, as they present differently, for the purpose of this study, information was obtained on common, generic strategies that are used, with three of the most common being: environmental modifications, compensatory strategies and direct remediation of skills, as recommended by Bellis (2003). The findings of the present study indicated that less than half of the participants provided intervention services. These finds were similar to that of the study conducted by Logue-Kennedy *et al.* (2011), where more than half of the study sample did not offer interventions in the area of APD and the minority who did indicate that their strategies only consisted of offering advice to the client, with no formal intervention protocol. Most of the participants offering intervention strategies in the form of advice were SLTs, their intentions being to manage underlying speech and language impairments rather than manage the APD itself (Logue-Kennedy et al., 2011). The United Kingdom has few referral systems in place for children with APD, despite the wide range of referring practitioners available (which includes parents; schools; ear, nose and throat specialists; SLTs and paediatricians).

Similar to the present study, audiologists in the study performed by Emanuel, Ficca & Korczak (2011) recommended a variety of strategies, including direct auditory training of listening skills, which was not a significant intervention practice in the present study. Despite several interventions services that are available to audiologists, the multidisciplinary team experiences challenges in reaching a consensus regarding the diagnosis. It is therefore recommended that in the interim, parents are provided with suggestions on how to support their children with APD (Slauterbeck, 2009). It can also be challenging for audiologists to engage with clients on a professional and ethical basis, if they do not have a clear understanding of their client’s cultural values and beliefs. Despite the fact that the emphasis on child healthcare is improving in South Africa, the country continues to follow Westernised, traditional, biomedical methods of assessment and intervention, which cannot always be effectively applied to the context of South Africa (Vaughn et al., 2009). The Westernised methods tend to believe that illness is often caused by natural influences, while the Africanised methods attribute illness and disease to supernatural forces or higher powers (Vaughn et al., 2009). Parents and caregivers alsoj need to understand the assessment and intervention programmes recommended by healthcare providers in order for them to fully invest into the programme (Popich, 2003). It can further be inferred that the identification and management of children with APD, particularly those living in poor socio-economic backgrounds, where intervention programmes are not deemed necessary by family members and caregivers, may prove to be challenging for the audiologist.

Audiologists attempting to provide services within the education sector in South Africa encounter many challenges due to a number of reasons, including the poor socio-economic conditions of their schools, their limited access to schools, overcrowded classrooms, poor teacher–child ratios and poor scholastic learning environments, to name a few (Department of Basic Education [DOE], 2010). The above-mentioned challenges are of particular concern for children already presenting with an APD or a learning disorder. Teachers are typically the first people to refer a child with APD to the audiologist, as also identified in the present study, yet are not always adequately equipped to handle the demands of the curriculum or address the requirements of learners with special education needs (Pottas, 2005). The challenges presented to the audiologist in attempting to facilitate team collaboration often results in a lack of carryover to the real-life context, such as the classroom. The above challenge may stem from a lack of awareness created amongst the team members and/or their training received in the area of APD, bringing into question the current quality of APD service delivery in South Africa.

The challenges presented in the current study are similar to those addressed in international studies. Whitelaw (2012) suggests that audiologists are frustrated by the lack of supporting documentation for the screening and assessment tools for APD, while Bellis (2003) suggests that audiologists may not wish to practice in the area of APD due to the time taken to screen, assess and diagnose the condition. According to the study conducted by Chermak *et al.* (2007), audiologists are unfamiliar with identifying which practitioners to refer to once a child had been screened for APD or once a diagnosis had been made. Whitelaw (2012) believes that audiologists avoid practicing in the area as they become despondent with the perception that APD cannot be cured. It is postulated that similar aspects are contributing factors as to why APD is not commonly managed in South Africa. The finding of this objective is concerning, as ethical and responsible practice is aligned to timeous and effective intervention strategies.

### Objective two

An open-ended question was included where the participants could provide recommendations with regards to managing children with APD. A total of 78 participants provided recommendations, which were categorised into five common themes and are presented below.

#### Reassessing the curricula of training audiologists/speech-language therapists and audiologists, and prioritising training at an undergraduate level

One of the common themes emerging from the current study was that the participants felt that there was little opportunity to manage children with APD during their undergraduate clinical training programme. Participants stated that they could have benefitted from additional theoretical coursework and clinical training. This may be achieved by allocating additional clinical hours to the area of APD. Therefore, a re-evaluation of the audiological undergraduate training curricula, with regard to APD, should be considered. One of the participants reported that:

‘Lecturers are not always equipped to deal with proper explanations to students therefore my training was insufficient’. (Audiologist, Bachelor’s degree, 0–5 years of experience)

#### Reinstating the South African Auditory Processing Disorder Taskforce to create standardised assessment tools, intervention strategies and policies

Another theme emerging from the current study was reinstituting the South African Taskforce, in order to develop standardised policies and guidelines. These guidelines may encourage confident training and practice among audiologists in the area of APD. A team steering the development of appropriate screening and assessment tools to suit the South African context, may improve the provision of APD services among audiologists in South Africa. The following was reported by one of the participants:

‘No golden standard/universal definition – so what exactly are we testing and managing? Pass-Fail criterion – some recommend 2 Standard Deviations (SD’s) and others 3 SD’s; so are our tests really sensitive enough for APD identification and diagnosis?’ (Audiologist, Master’s degree, based in academia)

As previously discussed, there are no single criteria by which APD screening and assessment results can be measured, due to the heterogeneity of the disorder and the network of childhood disorders often associated with APD. Previous results suggest that often audiologists avoid practicing in the area of APD, not only because of their perceived inadequate training provided during their undergraduate training programme but also due to the lack of standardised and contextually appropriate assessment tools available to South African audiologists. Upon personal observations, there appears to be a developing awareness amongst other practitioners, teachers and parents in the area of APD, as the numbers of referrals to the audiologist increase. However, contextually and linguistically appropriate assessment tools are still a concern. It can therefore be inferred that soon enough, South African audiologists may be placed in an ethical dilemma if the numbers of referrals from teachers and healthcare practitioners start to increase, whilst the paucity of contextually and linguistically inappropriate guidelines and assessment tools continues. It therefore becomes difficult for audiologists to determine the extent to which an APD exists, and the nature thereof. Assessment directs intervention, and if assessment is inappropriate or fails to take into account cultural and linguistic variability, then the results may be inaccurate and biased. Therefore, audiologists should consider the development and adaptation of assessment tools and procedures to meet the diverse needs of the population. Intervention should also be culturally appropriate and relevant for the population served (Pascoe & Norman, 2011).

#### Collaboration between the speech-language therapist and the audiologist

SLT assessment tools and the assessment tools administered by the audiologist should complement each other in order to determine whether an APD diagnosis exists (Bellis, 2003). Participants recommended that audiologists and SLTs work in cohesion with each other, rather than arguing the disorder from two different perspectives. One of the participants stated the following:

‘The speech therapy and audiology approaches to APD are so very different and my perception is that the SLT’s role is much better known and there are more test and assessment materials for the SLT management of APD, other than FM systems. I would recommend that the SLT be the main profession doing APD assessments and therapy’. (Audiologist, Bachelor’s degree in audiology, based within the hospital setting)

#### Creating awareness within the education system

Two smaller themes emerged from the study, with one being that awareness should be created among educators. The participants felt that children presenting with signs of APD are often referred to other practitioners first and are unaware of the audiologists’ role in the management of APD. Bellis (2003) emphasises the importance of early identification and the implications on the child’s academic and social development. Awareness created amongst teachers may therefore encourage the early identification of children at risk for an APD:

‘Main concerns are that other allied professionals are conducting management that is often eg. educational psychologists. They are even offering courses to train teachers and ignoring the scope of practice of the SLT and audiologist’. (Audiologist, Master’s degree, in academia setting)

#### CPD activities

The participants reported that attending additional workshops and courses providing training in the area of APD will help drive the management of APD in South Africa. The following was stated by one of the participants:

‘I attended a two day course led by Dr Wayne Wilson which was very helpful to understand APD and current issues. Such courses are needed to stay informed’. (Audiologist, Bachelor’s degree, in the school setting)

## Research implications

Creating standardised and reliable assessment tools that are culturally appropriate and linguistically suitable for South African children may aid the future development of the scope of APD and equip audiologists with the necessary tools to make an accurate diagnosis. Research projects should be dedicated towards developing, standardising and validating test materials. As the current study sample comprised audiologists and STAs only, very little information has been provided regarding the present practices of SLTs, specifically from a linguistic perspective. Similarities and/or discrepancies between the SLT and the audiologist will be able to offer rich information for future curricula development. The present study was also based on a quantitative paradigm. Future research using a mixed-method design of both qualitative and quantitative information may provide richer data with regards to the perspectives of audiologists in managing children with APD. An updated study on the current South African audiology training programmes would be beneficial in providing information where prioritisation is required for future training.

### Clinical implications

Reinstituting the South African Taskforce would encourage the development of new and updated policies and guidelines in South Africa, with regards to the present APD intervention trends. The taskforce could also serve as a lobbying body for resource allocation and awareness creation in the area of APD. This should include lobbying for additional human resources and training therapists from diverse backgrounds to address the cultural and linguistic issues and post creation. It is hoped that the present study will attract audiologists into the field of APD, should training workshops on a theoretical and practical level be supported. Educating medical aids and insurance companies on the present state of APD as a disorder, and motivating for the effective implementation of APD procedure codes, may shed light on effective practices in the area of APD.

### Limitations

A study sample comprised less than 10% of the study population and may therefore not be a true representation of South African audiologists. Information bias may also have occurred, as the responses obtained were dependent on the participants’ willingness to complete the research tool. Due to the lack of consensus with regards to the definition of APD and the training thereof, the participants’ responses were based on their own understandings of APD, and therefore, variable responses may be expected.
